# Equilibrium Dynamics of β-N-Methylamino-L-Alanine (BMAA) and Its Carbamate Adducts at Physiological Conditions

**DOI:** 10.1371/journal.pone.0160491

**Published:** 2016-08-11

**Authors:** David Zimmerman, Joy J. Goto, Viswanathan V Krishnan

**Affiliations:** 1 Department of Chemistry, College of Science and Mathematics, California State University, Fresno, California, 93740, United States of America; 2 Department of Medical Pathology and Laboratory Medicine, University of California Davis School of Medicine, Sacramento, California, 95817, United States of America; Weizmann Institute of Science, ISRAEL

## Abstract

Elevated incidences of Amyotrophic Lateral Sclerosis/Parkinsonism Dementia complex (ALS/PDC) is associated with β-methylamino-L-alanine (BMAA), a non-protein amino acid. In particular, the native Chamorro people living in the island of Guam were exposed to BMAA by consuming a diet based on the cycad seeds. Carbamylated forms of BMAA are glutamate analogues. The mechanism of neurotoxicity of the BMAA is not completely understood, and BMAA acting as a glutamate receptor agonist may lead to excitotoxicity that interferes with glutamate transport systems. Though the interaction of BMAA with bicarbonate is known to produce carbamate adducts, here we demonstrate that BMAA and its primary and secondary adducts coexist in solution and undergoes a chemical exchange among them. Furthermore, we determined the rates of formation/cleavage of the carbamate adducts under equilibrium conditions using two-dimensional proton exchange NMR spectroscopy (EXSY). The coexistence of the multiple forms of BMAA at physiological conditions adds to the complexity of the mechanisms by which BMAA functions as a neurotoxin.

## Introduction

The neurotoxicity of β-N-methylamino-L-alanine (BMAA) from the seeds of cycad plants was first demonstrated by Vega and Bell [1]. The higher than normal incidence of the Amyotrophic Lateral Sclerosis/Parkinsonism Dementia complex (ALS/PDC), particularly within the local Chamorro people of Guam, is associated with the presence of BMAA [2, 3]. The role of BMAA in the onset as well as progression of neurodegenerative diseases including Alzheimer's disease (AD) is strongly supported by many studies [4–9]. Based on the numerous *in vitro* investigations, the detrimental effects of BMAA are implicated due to the mechanism of excitotoxicity via the activation of excitatory amino acid (EAA) receptors [4, 10–14]. Excitotoxicity could be a significant contributor to neurodegenerative diseases as evidenced by the presence of increased levels of glutamate in the cerebrospinal fluid of ALS patients [15, 16].

The interaction of BMAA with bicarbonate ions is critical in the modality of BMAA’s role in excitotoxicity. Pioneering work by Weiss and Choi [17] demonstrated the requirement of bicarbonate ions as a cofactor for the activity of BMAA. All the assays performed since the discovery by Weiss and Choi [17], included bicarbonate ions (HCO_3_^-^) in the media at concentrations ranging from 10 mM to 25 mM [13]. The presence of bicarbonate ions at close to physiological pH therefore created ideal conditions for the formation of carbamate adducts [18]. Myers and Nelson [18] characterized the interaction between BMAA and the bicarbonate ions and identified the formation of β-carbamate of BMAA. These findings led to a high similarity between the chemical structures of β-carbamate of the BMAA and glutamic acid. This resemblance might hold the potential clue to the mechanism of how BMAA affects glutamate receptors [13, 17–20].

Molecular interactions with bicarbonate have been studied due to the importance of spontaneous carbamate formation in biological reactions. Carbamate reactions are reversible and occur as the result of a nucleophilic attack by uncharged amines on the carbon dioxide produced from bicarbonate, *in vivo* [21]. The exogenic reaction between the uncharged amine and carbon dioxide to form carbamates has considerable free energy contribution from the dissociation reaction involving carbamic acid (pK_a_ < 4.8) [22]. Under physiological conditions (pH ~7), the amines are mostly protonated (pK_a_ ~8) and the CO_2_ exists in the hydrated carbonic acid, H_2_CO_3_ (pK_a1_ ~6.3). These conditions tend to be unfavorable for carbamate formation at physiological conditions. Nonetheless the chemical environment can lower the pK_a_ of the protonated amines and thus benefit the carbamate formation which may be further stabilized by other intermolecular non-covalent interactions [23]. Carbamate formation plays an important role in many biological functions such as carboxylation of the active sites (lysine side chain ε-amino group), biosynthesis of biotin and purine biosynthesis [23–25], as well as methanogenic archaea mediated reduction of CO_2_ to methane [26]. The carbamate formation in most cases are non-enzymatic reactions as demonstrated in the case of the carbamate (N-carboxymethanofuran) in methanogenic archaea [24].

Nunn and co-workers first studied the changes in the NMR spectra of BMAA in the presence of bicarbonate ions and demonstrated the formation of the primary carbamate adduct [25, 26]. They also first hypothesized the formation of a second adduct of BMAA, but the presence was not demonstrated by NMR spectroscopy, as two-dimensional methods then were not routinely employed. In a later study by Myers and Nelson [18], ^13^C labeled bicarbonate was used to examine the formation of the BMAA/bicarbonate adducts. Myers and Nelson observed the formation of two carbamate adducts and found these products were formed when the amine, from the BMAA, binds to carbon dioxide produced from bicarbonate.

In this manuscript, we present a study of BMAA: HCO_3_^-^ interaction using high-resolution NMR spectroscopy. In addition to confirming the earlier observations that BMAA: HCO_3_^-^ interactions lead to the formation of both α and β carbamates, we observed that these adducts coexist in the solution state at physiological conditions. Proton chemical shifts of the carbamate adducts are distinctly different from the free BMAA and thus enable the characterization of the exchange kinetics. Two-dimensional saturation transfer exchange spectroscopy (EXSY) [27, 28] is used to determine the pseudo first order rate constants of the α- and β- carbamate formation/cleavage. The conformational preference of the carbamate adducts and the intermolecular dynamics may provide an important insight to the understanding of the neurotoxicity of BMAA.

## Materials and Methods

### Sample preparation

β-N-methylamino-L-alanine (BMAA): bicarbonate samples were prepared with a BMAA concentration of 5 mM with varying bicarbonate concentrations. HCO_3_^-^ concentrations were varied from 0.5 mM to 300 mM by adjusting the concentration of the added sodium bicarbonate in the solution. The BMAA:HCO_3_^-^ samples were prepared in 90% H_2_O and 10% D_2_O or in 100% D_2_O and pH (pD) adjusted to 7.6. A final volume of 600 μL is used for the NMR studies. In all the calculations the pH to pD conversion was not performed because of the constant term that relates pH to pD (Pd = pH+0.41) [29]. This is a valid approximation considering the decreased acidities of acids in D_2_O [30, 31].

### NMR spectroscopy

All NMR experiments were performed in a 400-MHz (^1^H resonance frequency) VNMRS spectrometer (Varian-Agilent). 2D EXSY spectra [27] were collected using standard NOESY pulse sequence in the phase-sensitive mode (States-TPPI). The probe temperature was 30°C. The mixing time for the EXSY experiment (τ_m_) was 400 ms to reduce the interference from zero-quantum induced coherence transfer between the J-coupled spins and other intramolecular NOE transfers [32]. Two-dimensional TOCSY experiment was performed with a DIPSI multiple pulse mixing sequence (mixing time 80 ms) [33]. All the 2D data were collected with 256 complex points along the indirect dimension (t_1_), 2048 complex points in t_2_ over a spectral width of 10 ppm and signal averaged over 32 transients with a recycle delay of 5 s. Time-domain data were zero-filled to 1024 points in ω_1_ and 4096 points in ω_2_. A 90°-shifted squared sine bell window function was applied prior to a Fourier transformation in each dimension. All the spectra were referenced to TSP (3-(Trimethylsilyl) propionic-2, 2, 3, 3-d_4_ acid sodium salt) signal at 0.0 ppm. One-dimensional NMR spectra of freshly prepared samples were performed before and after running the 2D experiments to confirm that the sample conditions were unaltered during the course of the data collection. The spectra were processed and analyzed using Mestrenova (Mestrelab Research, Santiago de Compostela, Spain).

### Chemical equilibrium dynamics of carbamate adduct formations

**Fig 1:**
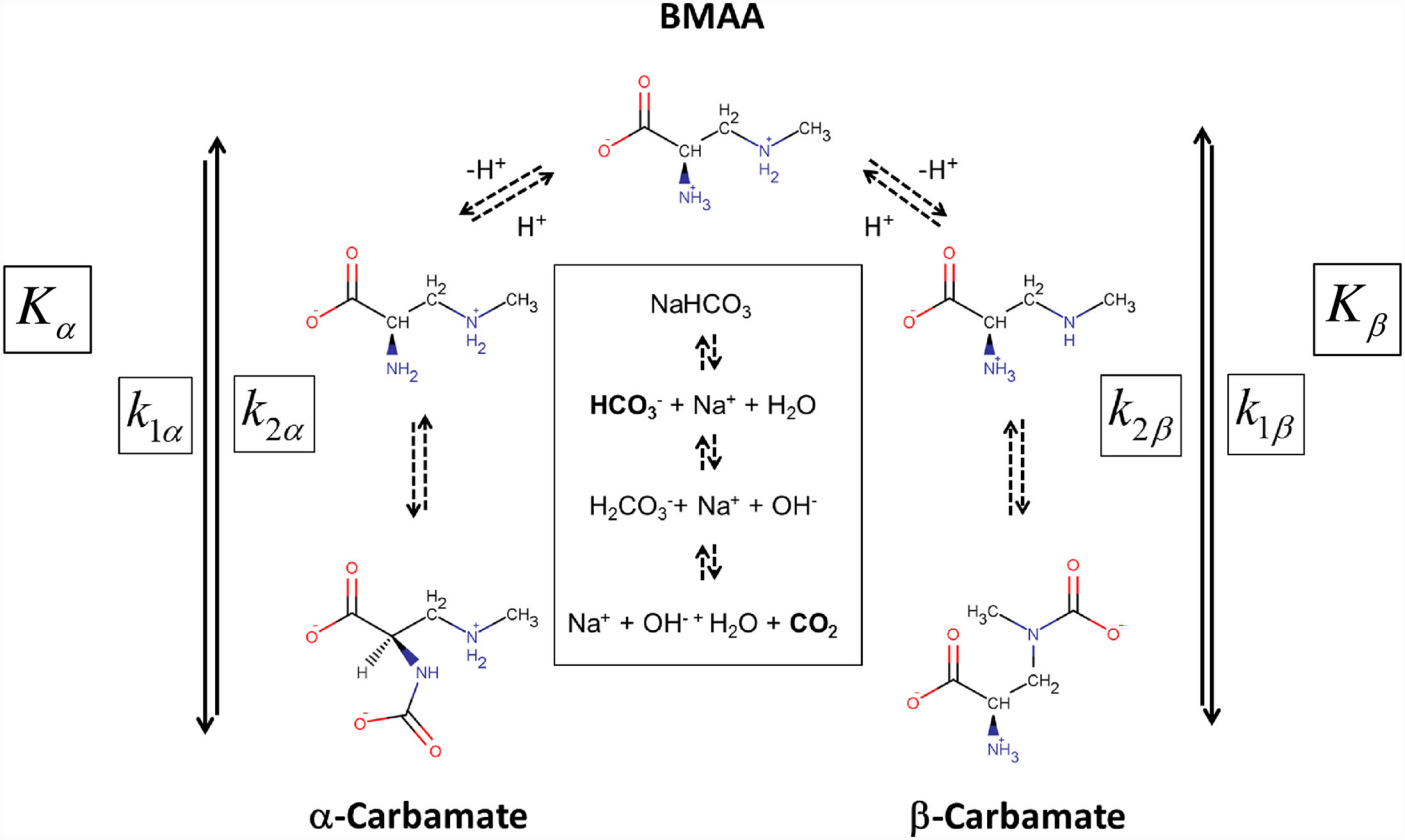
Kinetic equilibrium process of the conversion and chemical exchange of β-Methylamino-L-alanine (BMAA) with α-carbamate and β-carbamate upon interaction with sodium bicarbonate. At neutral pH both the α- and β- carbamate adducts coexist in solution and undergo detectable chemical exchange with the free BMAA. The exchange rates between the free BMAA and that of the α-carbamate and β-carbamates are defined as *k*_*1α*_ (reverse rate *k*_*2α*_) and *k*_*1β*_ (reverse rate *k*_*2β*_), with the chemical equilibrium constants of *K*_*α*_ and *K*_*β*_, respectively. Two-dimensional saturation transfer exchange spectroscopy (EXSY) is used to characterize the chemical exchange mechanism and equilibrium constants. Figure also shows the other reactions that are responsible for generating the bicarbonate ion and CO_2_ under aqueous conditions (middle part) and are shown in block letters. The two-way arrows with continuous lines (i.e., ⇄) to represent equilibria with kinetically measurable rate constants, while arrows with the dashed lines represent equilibrium processes that are faster than the NMR measured chemical exchange parameters.

The formation of α and β carbamate adducts of BMAA are shown in Fig 1, with an equilibrium constant of *K*_*α*_ and *K*_*β*_, respectively. The homogeneous interactions of CO_2_ in aqueous BMAA solutions include a complex series of reactions to form carbamates, hydration reactions involving hydroxide, water and CO_2_, as well as amines [34, 35]. Thus, the collection of reaction mechanisms involving the various ions and water must be accounted for and quantified to describe the chemical equilibrium of α- and β- carbamate formation (see Fig 1). In the presence of H^+^ and CO_2_, the formation of the α- and β- carbamates is assumed to follow the equilibrium constants defined as follows:

$$K_{\alpha }^{}=\frac{k_{1\alpha }}{k_{2\alpha }}=\frac{\left[\alpha -Carbamate\right]\left[H^{+}\right]}{{\left[BMAA\right]}_{}\left[CO_{2}\right]}\text{ and }K_{\beta }^{}=\frac{k_{1\beta }}{k_{2\beta }}={\frac{\left[\beta -Carbamate\right]\left[H^{+}\right]}{{\left[BMAA\right]}_{}\left[CO_{2}\right]}}_{}$$ (1)

**Fig 2:**
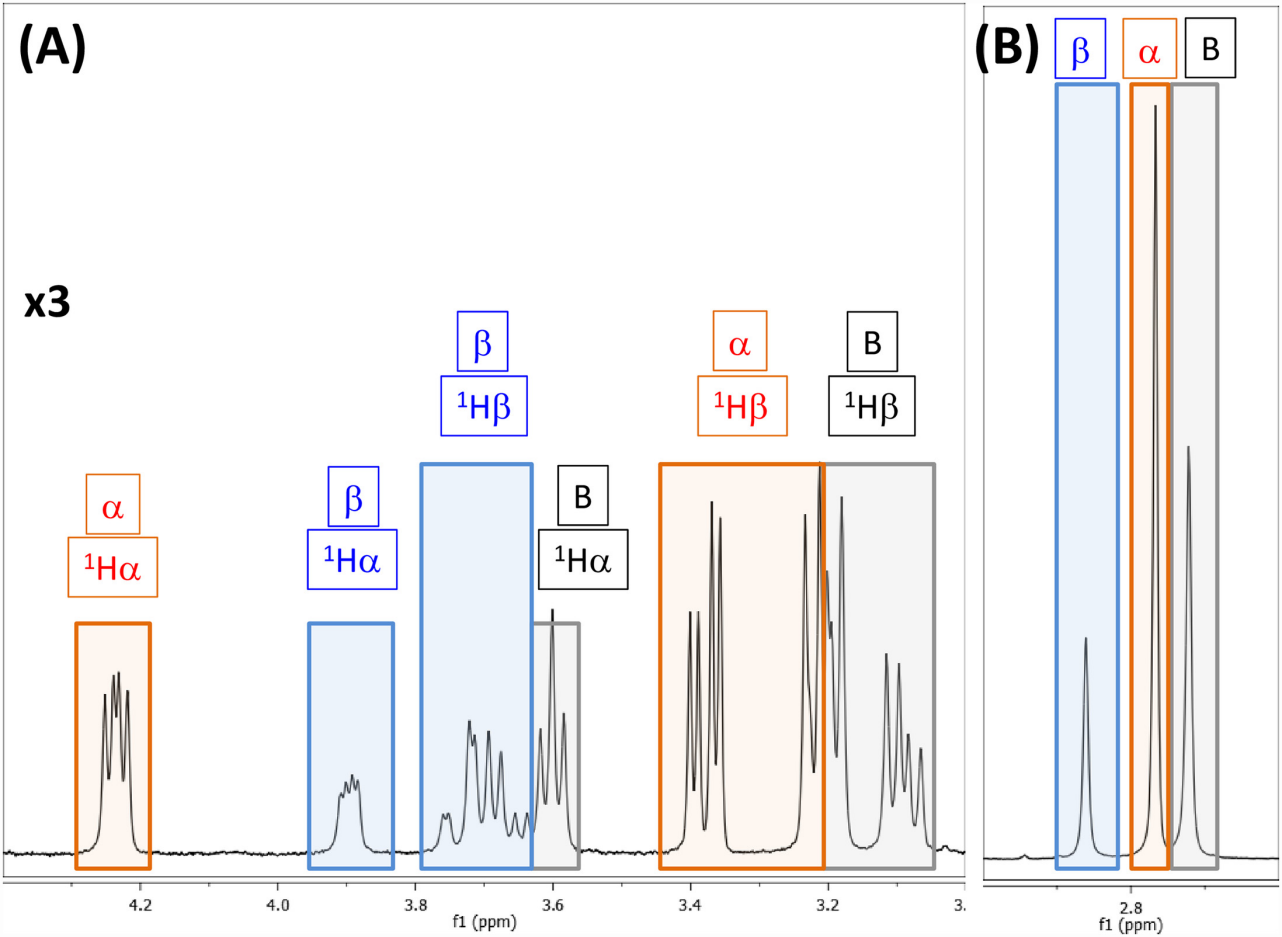
400 MHz ^1^H NMR spectrum of highlighting the co-existence of free BMAA (B: black boxes), α-carbamate adduct (α: red boxes) and β-carbamate adduct (β: blue boxes). The Hα and the Hβ protons are shown on the left (panel A) and the methyl protons on the right (panel B). NMR spectra were recorded with 1:20 ratio of BMAA: HCO_3_^-^ (10mM: 200mM) in 100% D_2_O, pD of 7.6 and at 30°C.

Where [α-carbamate] and [β-carbamate] are the concentrations of the α- and β- carbamates in equilibrium with the BMAA ([BMAA]) and carbon dioxide ([CO_2_]). Based on the NMR data (Fig 2) and the previous studies [18, 25] there is no evidence of the formation of αβ-carbamate (doubly carbamylated) form of BMAA and is therefore not considered as part of the reaction mechanism.

In order to determine the equilibrium constants (Eq (1)), all the concentration values are required. However, the NMR spectrum cannot differentiate between the protonated vs. deprotonated forms of the carbamate adducts (Fig 1) and therefore all the BMAA concentrations must be related to the starting total BMAA concentration ([BMAA]_T_). During the chemical exchange process, the total BMAA concentration is

$${\left[BMAA\right]}_{T}=\left[BMAA\right]+\left[BMAA-\alpha^{+}\right]+\left[BMAA-\beta^{+}\right]$$ (2)

Where [BMAA-α^+^] and [BMAA-β^+^] are the concentrations of BMAA protonated at the primary and secondary amines, respectively (Fig 1). The equilibrium process of the adduct formation will depend on the total amount of carbon dioxide present in the experimental vessel. The total concentration of CO_2_ ([CO_2_]_T_) can also be defined in terms of the various reactions (Fig 1, center) as

$${\left[\text{C}{\text{O}}_{2}\right]}_{T}=\left[CO_{2}\right]+\left[H_{2}CO_{3}^{-}\right]+\left[HCO_{3}^{-}\right]+\left[CO_{3}^{2-}\right]$$ (3)

It is important to note the integral role of bicarbonate ion in Eq (3) (Fig 1) in the formation of CO2 [25, 26, 36]. Using the definitions of total concentrations of BMAA and CO_2_ from Eqs (2) and (3), the equilibrium process for the formation of carbamate adducts can be written as

$$K_{\alpha }^{*}=\frac{k_{1\alpha }^{*}}{k_{2\alpha }^{*}}=\frac{\left[\alpha -Carbamate\right]}{{\left[BMAA\right]}_{T}{\left[CO_{2}\right]}_{T}}\text{ and }K_{\beta }^{*}=\frac{k_{1\beta }^{*}}{k_{2\beta }^{*}}=\frac{\left[\beta -Carbamate\right]}{{\left[BMAA\right]}_{T}{\left[CO_{2}\right]}_{T}}$$ (4)

Where *K*_α_* and *K*_β_* are the equilibrium constants involving the total concentrations of BMAA and CO_2_. Eq (4) is the representation of Eq (1) in terms of the total concentration of BMAA and CO_2_. In Eq (4), *k*_1i_* and *k*_2i_* with *i* = α or β are defined as

$$k_{1i}^{*}=k_{1i}\frac{\left[BMAA\right]\left[CO_{2}\right]}{{\left[BMAA\right]}_{T}{\left[CO_{2}\right]}_{T}}\text{ and }k_{2i}^{*}=k_{2i}\left[H^{+}\right]$$ (5)

Defining *k*_1i_** = *k*_1i_*[*CO*_*2*_]_*T*_ (*i = α or* β), the equilibrium constants of the adduct formation (Eq (4)) can be written as

$$K_{\alpha }^{**}=\frac{k_{1\alpha }^{**}}{k_{2\alpha }^{*}}=\frac{\left[\alpha -Carbamate\right]}{{\left[BMAA\right]}_{T}}\text{ and }K_{\beta }^{**}=\frac{k_{1\beta }^{**}}{k_{2\beta }^{*}}=\frac{\left[\beta -Carbamate\right]}{{\left[BMAA\right]}_{T}}$$ (6)

The constants *k*_1i_** and *k*_2i_* (*i = α or* β) (i = α or β) are the pseudo first order exchange constants of carbamate adduct formation and pseudo first order exchange constant of carbamate cleavage, respectively.

The total concentration of BMAA (Eq (2)) can be estimated using the pK_a_ of protonated and non-protonated amines. Similarly, the total concentration of the carbon dioxide is estimated using the equilibration process of carbamate and carbamic acid. Two-dimensional EXSY spectrum is used to determine the rate constants *k*_1α_**, *k*_2α_* (and *k*_1β_**, *k*_2β_*) (Eqs (4) and (6)), while using the equilibrium concentrations of [CO_2_]_T_, [CO_2_] and [H^+^]. It must be noted that these calculations are valid at any given sample conditions (BMAA:HCO_3_^-^ concentration). In the event of concentration dependent change in the equilibrium, this approach measures the *quasi-equilibrium* kinetics.

### Calculation of exchange rates from EXSY data

The equilibrium process between the BMAA and its α- and β- carbamates affects the nuclear spin relaxation process via the mechanism of chemical exchange. For a system of three spins (I = ½), the nuclear spin relaxation process in terms of the rate constants of chemical kinetics can be written in terms of the relaxation matrix as [27, 28, 37]

$$\tilde{A}=\text{exp}\left(-\tilde{R}\tau_{m}\right).$$ (7)

where τ_m_ is the mixing time used in the EXSY experiment and the elements of *Ã* (matrix) are defined as [27, 28]

$${\tilde{A}}_{ij}=\frac{V_{ij}}{p_{j}},$$ (8)

with ‘i’ and ‘j’ are the three chemically exchanging sites from three molecules: free BMAA, α- carbamate and β-carbamate adducts. V_ij_ are the cross peak volumes between chemical shifts of ‘i’ and ‘j’ and *p*_*j*_’s are the relative population of the i^th^ species. The relative populations of the three molecules can be defined as

$$p_{{\left[BMAA\right]}_{T}}={\left[BMAA\right]}_{T}/\left({\left[BMAA\right]}_{T}+\left[\alpha -Carbamate\right]+\left[\beta -Carbamate\right]\right)$$ (9)

$$p_{\left[\alpha -Carbamate\right]}=\left[\alpha -Carbamate\right]/\left({\left[BMAA\right]}_{T}+\left[\alpha -Carbamate\right]+\left[\beta -Carbamate\right]\right)$$ (10)

$$p_{\left[\beta -Carbamate\right]}=\left[\beta -Carbamate\right]/\left({\left[BMAA\right]}_{T}+\left[\alpha -Carbamate\right]+\left[\beta -Carbamate\right]\right)$$ (11)

Using the definitions from Eqs (8)–(11), the matrices *Ã* and *Ř* in Eq (7) can be written as

$$\tilde{A}=\left(\begin{array}{ccc} \frac{V_{{\left[BMAA\right]}_{T},{\left[BMAA\right]}_{T}}}{p_{{\left[BMAA\right]}_{T}}} & \frac{V_{{\left[BMAA\right]}_{T},\left[\alpha -Carbamate\right]}}{p_{\left[\alpha -Carbamate\right]}} & \frac{V_{{\left[BMAA\right]}_{T},\left[\beta -Carbamate\right]}}{p_{\left[\beta -Carbamate\right]}}\\ \frac{V_{\left[\alpha -Carbamate\right],{\left[BMAA\right]}_{T}}}{p_{{\left[BMAA\right]}_{T}}} & \frac{V_{\left[\alpha -Carbamate\right],\left[\alpha -Carbamate\right]}}{p_{\left[\alpha -Carbamate\right]}} & \frac{V_{\left[\alpha -Carbamate\right],\left[\beta -Carbamate\right]}}{p_{\left[\beta -Carbamate\right]}}\\ \frac{V_{\left[\beta -Carbamate\right],{\left[BMAA\right]}_{T}}}{p_{{\left[BMAA\right]}_{T}}} & \frac{V_{\left[\beta -Carbamate\right],\left[\alpha -Carbamate\right]}}{p_{\left[\alpha -Carbamate\right]}} & \frac{V_{\left[\beta -Carbamate\right],\left[\beta -Carbamate\right]}}{p_{\left[\beta -Carbamate\right]}} \end{array}\right)\;$$ (12)

$$\;\tilde{R}=\left(\begin{array}{ccc} R_{BMAA}+k_{1\alpha }^{**}+k_{1\beta }^{**} & -k_{2\alpha }^{*} & -k_{2\beta }^{*}\\ -k_{1\alpha }^{**} & R_{\alpha }+k_{2\alpha }^{*} & -k_{2\alpha \beta }^{*}\\ -k_{1\beta }^{**} & -k_{1\beta \alpha }^{*} & R_{\beta }+k_{2\beta }^{*} \end{array}\right)$$ (13)

The relaxation matrix *Ř* contains the kinetic parameters of the chemical exchange and longitudinal relaxation rates. In matrix *Ã*, the quantities *V*_*ij*_ *(i*, *j = [BMAA]*_*T*_, or *[α-carbamate]* or *[β-carbamate]*) are the two-dimensional cross-peak volumes in the EXSY experiment and *p*_*i*_ *([BMAA]*_*T*_, *or [α-carbamate] or [β-carbamate]*) are the relative populations determined from the 1D-NMR spectrum obtained at the same conditions (or from the diagonal peak volumes of the EXSY spectrum (*V*_*ij*_)). The rate constants *k*_1βα_* and *k*_2αβ_* are the magnetization transfer resulting from the coupled linear differential equation relating non-equilibrium nuclear spin magnetization. These second order (relayed) transfer of magnetization rates are included in the calculations to account that the total magnetization within the exchanging spins is conserved. The *Ř* matrix can be obtained by diagonalizing *Ã* as

$$\tilde{R}=-\frac{\text{ln}\left(\tilde{A}\right)}{\tau_{m}}=-\frac{\hat{U}\left(\text{ln }\tilde{\Lambda }\right){\hat{U}}^{-1}}{\tau_{m}}$$ (14)

Where *Û* is the square matrix of eigenvectors of *Ã*, such that *Û*^*-1*^ *Ã Û = λ = diag (λ*_*j*_*)* and *ln(Λ) = diag (ln λ*_*j*_*)*, with *λ*_*j*_ the j^th^ eigenvalue of *Ã* [37–39]. Eq (13) can be solved by several standard routines and herein we have used “R-Statistical Programming” for the process [40].

The concentrations of [BMAA]_T_, [α-carbamate] and [β-carbamate]) in the 600 μL reaction vessels (NMR tubes) were obtained from the integration of the 1D spectra (total BMAA concentration = [BMAA]_T_, or [α-carbamate] or [β-carbamate]). Concentration of BMAA (unprotonated) were calculated using the known pK_a_ of amines [41]. We further assume that the pK_a_ for primary and secondary amines are the same and temperature independent. Total carbon dioxide concentration was estimated using the total NaHCO_3_ in the solution (pK_a1_ of H_2_CO_3_ = 6.34) [42]. At equilibrium conditions close to the neutral pH, the ratios [H_2_CO_3_]/[CO_2_] and [H_2_CO_3_]/[HCO_3_^-^] are small (< 0.005 or less) [43]. Furthermore, contributions from CO_3_^2-^ may also be neglected, as the pK_a2_ of H_2_CO_3_ is 10.3. Upon substituting these values and the corresponding rate constants determined from the EXSY spectrum, the equilibrium constant can be determined in a straightforward manner.

Uncertainty in the measured values were determined by error propagation method (using R). The standard deviation in the spectral data was measured by estimating the noise in the 1D or 2D data by randomly selecting five different regions of the spectra. The standard deviation in each of the measured values was simulated randomly using a *Monte Carlo* method (5000 samples) with respect to the mean and standard deviation of the of the individual variables in the respective measurements. Typically, the accuracy of the measured peak intensity in the 1D spectra is ~1% and the variation in the diagonal and cross peak volumes of the 2D data are in the range of 3–7%. The details of the calculations, in spreadsheet format along with the R-code, used for matrix diagonalization are provided in S1 Table.

## Results

### NMR characterization of the BMAA-carbamate adducts

Proton NMR spectra of BMAA in the presence of bicarbonate ions show spectral features from the free BMAA and the α- and β- carbamate adducts (Fig 2). The NMR spectra of BMAA and carbamates are similar to the NMR results obtained by Nunn and co-workers [25, 26] except for the increased spectral resolution (^1^H frequency at 400 MHz vs. 300 MHz). We did not find any evidence of the presence of doubly carbamylated form of BMAA in the NMR spectra, which is consistent with the previous observations [18, 25]. The spectral patterns of all the protons are distinctly different between the three molecules in terms of chemical shift and coupling constants. At 400 MHz, the two ^1^Hβ protons and ^1^Hα proton show a typical ABX spin-system with the ^1^Hα proton chemical shifts of the α-carbamate, β-carbamate and the free BMAA at 4.24 ppm (dd), 3.90 ppm (dd) and 3.6 ppm (t), respectively. The AB part of the ABX spin system represented by the ^1^Hβ spins are also distinctly different for the three molecules (Fig 2, Panel A). The methyl resonances of the free BMAA, α-carbamate and β-carbamate are at 2.72 ppm, 2.77 ppm and 2.86 ppm, respectively (Fig 2, Panel B). Using the intensity of the methyl groups the free BMAA, α-carbamate and β-carbamate molecules are distributed by 33%, 46% and 21%, respectively (BMAA:HCO_3_ = 1:20 and at 303 K). The relative distribution of the three molecules changes with increasing concentration of the HCO_3_^-^ (see below).

Two-dimensional total correlation spectroscopy (TOCSY) experiment was used to identify the sub-set of the intramolecular J-coupled correlations with the BMAA, α-carbamate and β-carbamate molecules (S1 Fig**)**. Free BMAA in equilibrium with the two BMAA carbamate adducts was established by the two-dimensional EXSY experiment (S2 Fig). The EXSY experiment shows saturation transfer of magnetization between the protons that undergo chemical exchange between the free-BMAA, and its α- and β-carbamate adducts. The free-BMAA peaks are identified in comparison with the NMR spectrum of the BMAA in absence of bicarbonate ions (data not shown). The β-carbamate formation brings the carbonyl oxygen in closer vicinity to the methyl group (Fig 1) leading to a downfield shift. Therefore, the methyl resonance at 2.86 ppm is assigned to the β-carbamate, confirming the earlier observation by Nunn et al. [25, 26]. The chemical shift assignments for rest of the protons are consistent with the cross peak patterns observed in the TOCSY experiment (S1 Fig).

### Equilibrium dynamics of BMAA: adducts

**Fig 3:**
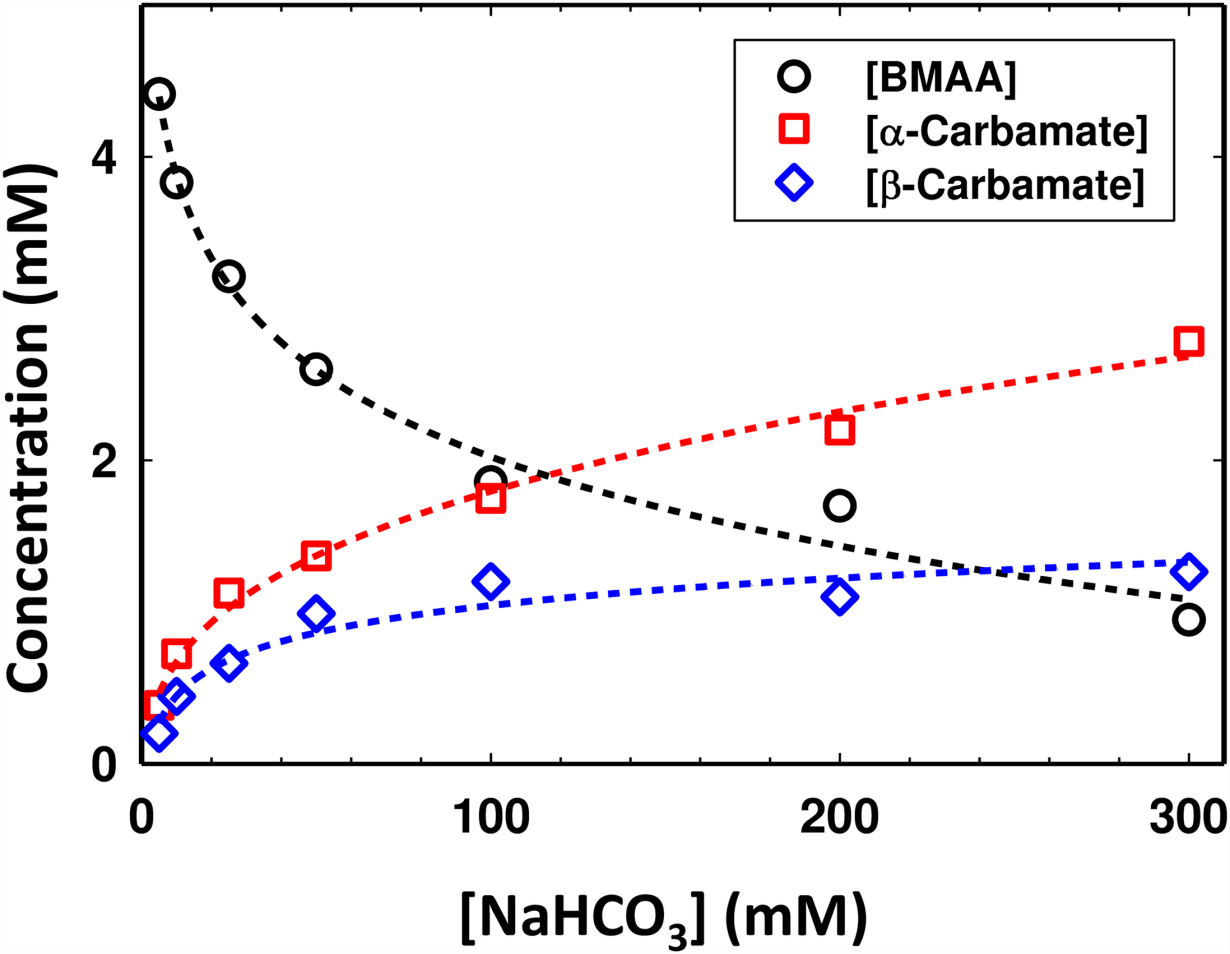
Formation of α-carbamate and β-carbamate in the equilibrium process and its dependence of concentration of bicarbonate ions in the solution. Estimation of the total concentration of different species in solution [BMAA] (black circles), [α-carbamate] (red squares) and [β-carbamate] (blue diamonds) as a function of increasing HCO_3_^-^ ([NaHCO_3_]) concentration. The concentrations of each species were determined using one-dimensional ^1^H NMR spectroscopy. The concentration of the initial BMAA was 5 mM and all the experiments were performed at a pD of 7.6 and at 30°C.

The equilibrium process of the carbamate formation is slow and depends on the relative concentrations of the BMAA to HCO_3_^-^ ions. Fig 3 shows the variation in the amount of carbamates (α and β) formed as a function of increasing bicarbonate ions for a fixed concentration of BMAA (5 mM). The continuous curves are polynomial fit to the experimental points and may only be used to suggest an expected trend. As seen in Fig 3, even at a BMAA: HCO_3_^-^ of 1:30, there is a significant equilibrium dynamic process between the free BMAA and the α- and β- carbamates.

**Fig 4:**
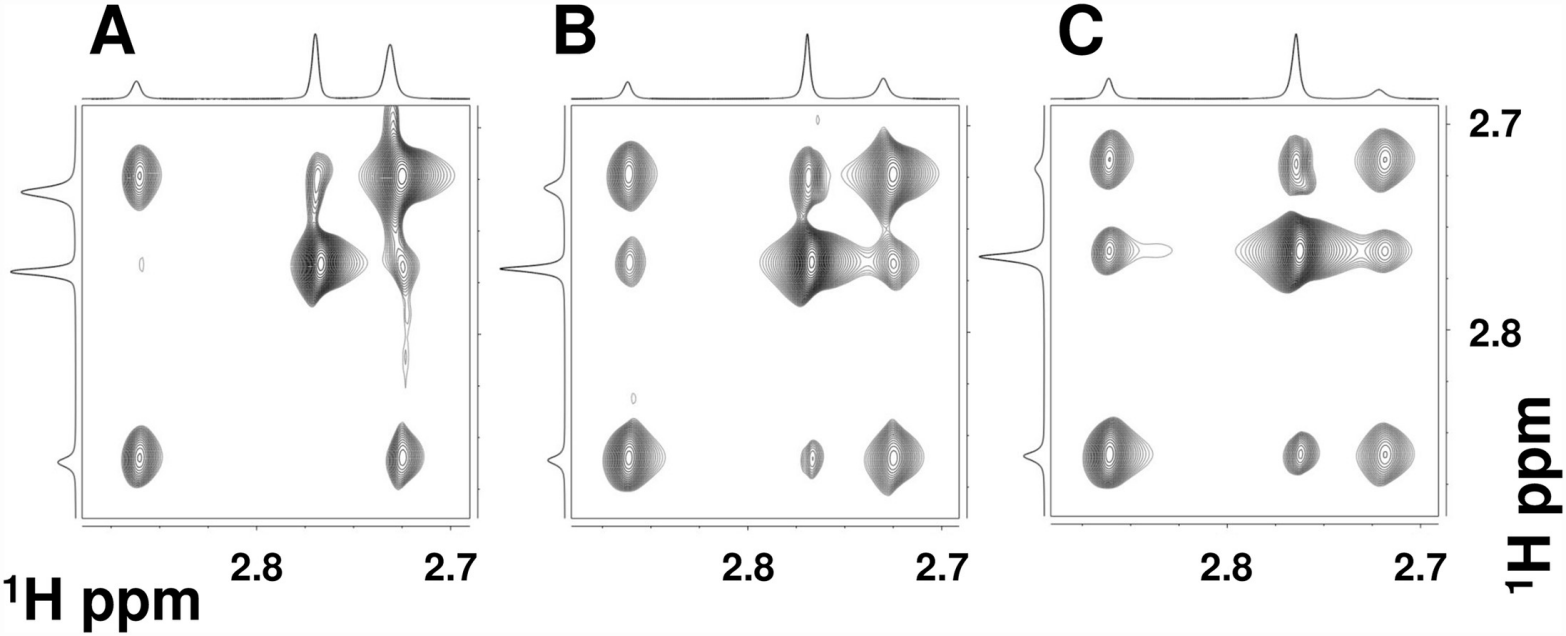
Effect of increasing bicarbonate concentration: Methyl region of the 400 MHz two-dimensional exchange spectroscopy (EXSY) spectra with increasing BMAA: HCO3-ratio: (A) 1:10, (B) 1:20 and (C) 1:30. The BMAA concentration was at 10 mM. EXSY spectrum is recorded at 30°C and with a mixing time of 400 ms.

All the resonances (^1^Hα, ^1^Hβ and ^1^Hγ) from the BMAA, the α-carbamate, and the β-carbamate show signs of chemical exchange between the three forms (S2 Fig). The methyl proton (^1^Hγ) resonances of the three molecules are void of overlap from other coherence transfer peaks (Fig 2 Panel B, S2 Fig) and therefore this region can be used to determine the kinetic parameters. The chemical exchange between the three species in the solution is reflected in the two-dimensional EXSY experiment between the methyl protons (Fig 4). The relative intensity of the cross peaks suggests that BMAA to α-carbamate exchange vs. the BMAA to β-carbamate exchange mechanisms are expected to have different rates. The cross peaks between the α- and β- carbamates at higher BMAA to HCO_3_^-^ are due to relayed transfer effects and are expected to increase in intensity particularly at longer mixing times of the EXSY experiments. The magnetization must be along the z-axis during the mixing period of the EXSY experiment. This allows for the exchange process between the various non-equilibrium spin states. In addition to the chemical exchange process, both cross-relaxation (NOE effects) and coherence transfer via zero quantum coherences, between the J-coupled spins, are active. The NOE contributions might underestimate the exchange contributions particularly for the small molecules and zero-quantum coherence transfer peaks that have anti-phase dispersive components in the spectra [44]. One of the effective methods to reduce the effects of both cross relaxation and zero-quantum induced transfers, particularly in the EXSY experiment, is to perform the EXSY experiments with relatively long mixing times. The EXSY experiments here are performed with mixing times in the range of 300–700 ms (data not shown) and found the mixing a time of 400 ms provided optimum sensitivity.

For a given concentration of BMAA and bicarbonate ions, the equilibrium constant between the BMAA to the carbamate adducts can be estimated using the EXSY data (Materials and Methods). Using a BMAA:HCO_3_^-^ ratio of 1:10 (BMAA concentration, 5 mM) from the one-dimensional ^1^H spectra (methyl groups) and the cross peak intensities of the 2D- EXSY spectra the relative populations of total BMAA (*p*_*[BMAA]T*_, Eq (9)), α-carbamate adduct (*p*_*[α-BMAA]T*_, Eq (10)) and β-carbamate adduct (*p*_*[β-BMAA]T*_, Eq (11)) are estimated to be 47%, 39% and 14%, respectively (Fig 4, S1 Table). The corresponding matrices *Ã* (Eq (12)),

$$\begin{array}{lll} \tilde{A} & = & \left(\begin{array}{ccc} \frac{V_{{\left[BMAA\right]}_{T},{\left[BMAA\right]}_{T}}}{p_{{\left[BMAA\right]}_{T}}} & \frac{V_{{\left[BMAA\right]}_{T},\left[\alpha -Carbamate\right]}}{p_{\left[\alpha -Carbamate\right]}} & \frac{V_{{\left[BMAA\right]}_{T},\left[\beta -Carbamate\right]}}{p_{\left[\beta -Carbamate\right]}}\\ \frac{V_{\left[\alpha -Carbamate\right],{\left[BMAA\right]}_{T}}}{p_{{\left[BMAA\right]}_{T}}} & \frac{V_{\left[\alpha -Carbamate\right],\left[\alpha -Carbamate\right]}}{p_{\left[\alpha -Carbamate\right]}} & \frac{V_{\left[\alpha -Carbamate\right],\left[\beta -Carbamate\right]}}{p_{\left[\beta -Carbamate\right]}}\\ \frac{V_{\left[\beta -Carbamate\right],{\left[BMAA\right]}_{T}}}{p_{{\left[BMAA\right]}_{T}}} & \frac{V_{\left[\beta -Carbamate\right],\left[\alpha -Carbamate\right]}}{p_{\left[\alpha -Carbamate\right]}} & \frac{V_{\left[\beta -Carbamate\right],\left[\beta -Carbamate\right]}}{p_{\left[\beta -Carbamate\right]}} \end{array}\right)\\ & = & \left(\begin{array}{ccc} 22.93 & 2.79 & 14.50\\ 2.10 & 36.08 & 1.36\\ 4.58 & 0.80 & 17.78 \end{array}\right) \end{array}$$ (15)

And *Ř* (Eq (14)) become

$$\;\tilde{R}=\left(\begin{array}{ccc} R_{BMAA}+k_{1\alpha }^{**}+k_{1\beta }^{**} & -k_{2\alpha }^{*} & -k_{2\beta }^{*}\\ -k_{1\alpha }^{**} & R_{\alpha }+k_{2\alpha }^{*} & -k_{2\alpha \beta }^{*}\\ -k_{1\beta }^{**} & -k_{1\beta \alpha }^{*} & R_{\beta }+k_{2\beta }^{*} \end{array}\right)=-\left(\begin{array}{ccc} 7.62 & 0.23 & 1.90\\ 0.17 & 8.96 & 0.07\\ 0.59 & 0.05 & 6.95 \end{array}\right)$$ (16)

The noise level in the 1D and 2D EXSY spectra are estimated from the regions of the spectra that do not have any peaks to be < 1% and 3–7%, respectively. The *Ř* (units of s^-1^) shows the exchange dynamics between the three species in solution. At the ratio of BMAA: HCO_3_^-^ (1:10), the rate of formation of the α-carbamate and β-carbamate adducts are 0.17 ± 0.03 s^-1^ (*k*_1α_**) and 0.60 ± 0.05 s^-1^ (*k*_1β_**), while the cleavages are 0.23 ± 0.02 s^-1^ (*k*_2α_*) and 1.89 ± 0.03 s^-1^ (*k*_2β_*), respectively (from matrix elements in Eq (16)). The half-lives of the formation the α-carbamate and β-carbamate adducts (assuming a first order reaction) are 4.18 ± 0.99 s (*t*_*½*,*1α*_) and 1.17 ± 0.10 s (*t*_*½*,*1β*_), while the cleavages are 3.08 ± 0.34 s (*t*_*½*,*2α*_) and 0.37 ± 0.01 s (*t*_*½*,*2β*_), respectively. The half-life of the α-carbamate formation decreases with increasing bicarbonate formation with increasing carbonate ion concentration while the cleavage increases (Table 1) suggesting that the system is still in *quasi-equilibrium* condition. Indirect (relayed) magnetization transfer rates between the adducts (*k*_1βα_* and *k*_2αβ_*) are much smaller than the direct transfer terms. Upon converting the exchange rates as described previously (Materials and Methods, S1 Table), the equilibrium constant (*K*_i_*, Eq (4)) for the formation of α-carbamate and β-carbamate adducts are estimated as 7.70 ± 1.77 and M^-1^ and 3.11 ± 0.26 M^-1^, respectively.

**Table 1:** Equilibrium rate constants of BMAA with α-carbamate and β-carbamate adducts using EXSY experiments.

	BMAA:HCO_3_^-^ = 1:10		BMAA:HCO_3_^-^ = 1:20		BMAA:HCO_3_^-^ = 1:30	
Kinetic Parameters (i = α or β)	α-carbamate	β-carbamate	α-carbamate	β-carbamate	α-carbamate	β-carbamate
$k_{1i}^{**}\;\left(s^{-1}\right)$	0.17 ± 0.03	0.6 ± 0.05	0.45 ± 0.02	1.13 ± 0.03	0.85 ± 0.03	1.66 ± 0.04
t12,1i (s)	4.18 ± 0.99	1.17 ± 0.1	1.53 ± 0.07	0.61 ± 0.01	0.82 ± 0.07	0.42 ± 0.08
$k_{1i}^{*}\;\left(M^{-1}s^{-1}\right)$	1.73 ± 0.35	5.97 ± 0.49	2.27 ± 0.1	5.65 ± 0.13	2.83 ± 0.1	5.53 ± 0.01
*k*_1_(*M*^-1^*s*^-1^)	0.04 ± 0.01	0.15 ± 0.01	0.06 ± 0.01	0.14 ± 0.02	0.07 ± 0.01	0.14 ± 0.01
$k_{2i}^{*}\;\left(s^{-1}\right)$	0.23 ± 0.02	1.89 ± 0.03	0.17 ± 0.02	1.75 ± 0.02	0.14 ± 0.05	1.25 ± 0.15
t12,2i (s)	3.08 ± 0.34	0.37 ± 0.01	4.13 ± 0.49	0.4 ± 0.05	5.02 ± 0.14	0.55 ± 0.11
*k*_2*i*_(*M*^−1^*s*^−1^) × 10^−8^	5.72 ± 0.62	47.59 ± 0.65	4.27 ± 0.49	43.91 ± 0.52	3.47 ± 0.2	31.5 ± 0.12
$K_{i}^{*}\;\left(M^{-1}\right)$	7.7 ± 1.77	3.15 ± 0.26	13.52 ± 1.71	3.23 ± 0.09	20.51 ± 0.58	4.41 ± 0.2
*K*_*i*_ × 10^6^	7.6 ± 1.75	3.11 ± 0.26	13.35 ± 1.69	3.19 ± 0.08	20.25 ± 0.58	4.36 ± 0.09
ΔGi* (kJmol)	-5.14 ± 1.18	-2.89 ± 0.24	-6.56 ± 0.83	-2.96 ± 0.08	-7.61 ± 0.22	-3.74 ± 0.17

For each i = α (α-carbamate) and i = β (β-carbamate) the rate constants $k_{1i}^{**}$ and $k_{2i}^{*}$ were determined by proton exchange NMR spectroscopy (EXSY). Reaction half-lives were determined by assuming a first order kinetics (t12,i=ln(2)ki). The equilibrium constants $K_{i}^{*}$ were then calculated using $k_{1i}^{*},k_{1i}$ and *k*_2*i*_ along with the estimates obtained by propagating the measured standard error in the NMR spectra (materials and methods). The Gibbs free energy was then estimated using $\Delta G_{i}^{*}=-RT\text{ln}\left(K\right)$. The experiments were performed in a total volume of 600 μL containing 5 mM BMAA and varying concentration of NaHCO_3_ (100 mM, 200 mM and 300 mM). The sample pD was at 7.6 and the experiments were performed at 303 K.

A summary of the experimentally measured equilibrium constants of the carbamate adduct formation is given (Table 1) along with the other relevant first and second order rate constants. A spreadsheet describing the calculations along with an R-code for matrix diagonalization is provided in S1 Table. To account for the protonation (*K*_*i*_, Eq (1)), the equilibrium constants of the formation of α-carbamate and β-carbamate are 7.60 ± 1.75 ×10^6^ and 3.11 ± 0.26×10^6^, respectively. Using the equilibrium conditions, the reaction Gibbs free energy (Δ*G*_α_*) for the α-carbamate formation was estimated to be -5.14 ± 1.18 kJ mol^-1^, and -2.89 ± 0.24 kJ mol^-1^ for the β-carbamate (Table 1). By increasing the concentration of the bicarbonate ions to 200 mM (BMAA:HCO_3_^-^ ratio of 1:20) the concentration of CO_2_ increases, which leads to an increase of *K*_1α_* to 13.52 ± 1.71 M^-1^ and *K*_1β_* to 3.23 ± 0.09 M^-1^ and the Gibbs free energy decreases (Δ*G*_α_* = -6.56 ± 0.83 kJ mol^-1^ and Δ*G*_β_* = -2.96 ± 0.08 kJ mol^-1^). The trend continues further upon increasing the concentration of NaHCO_3_ to 300 mM (BMAA: HCO3^-^ ratio of 1:30) with *K*_1α_* = 20.51 ± 0.58 M^-1^, Δ*G*_α_* = -7.61 ±0.22 kJ.mol^-1^, *K*_1β_* = 4.41 ± 0.20 M^-1^ and Δ*G*_β_* = -3.74 ± 0.17 kJ.mol^-1^ (Table 1). A linear trend between the equilibrium constants and the total concentration of NaHCO_3_ is observed: *K*_1α_* increases approximately 0.07 M^-1^ for an increase of 1 mM of HCO_3_^-^ ion, while *K*_1β_* increases approximately by 0.006 M^-1^ per 1 mM of HCO_3_^-^.

## Discussion

Carbamate synthesis in biological systems has an integral dependence on the presence of non-protonated form of the amines, which are the preferred protonation states under alkaline conditions. Aerial carbonation of amides to form carbamates is part of the biological fixation of CO_2_ by many organisms. Carbamate formation has been implicated in many biological functions ranging from inflammatory responses to tumor progression [45–50]. In particular, the formation of α-carbamate by cysteine and its neurotoxicity is bicarbonate-dependent [51].

Erving et al. have studied the rate of single carbamate formation in aqueous solution using spectrophotometric methods, including NMR spectroscopy [21]. EXSY based determination of both carbamate formation (*k*_*1α*_ and *k*_*1β*_) and cleavage (*k*_*2α*_ and *k*_*2β*_) are within the range of experimental measurements of aliphatic amines [24]. Similar results have been obtained in an EXSY based approach for single carbamate formation in N-carboxymethanofuran (carbamate) from methanofuran [24]. The measured experimental equilibrium constant for the β-carbamate (*K*_β_* = *k*_1β_*/ *k*_2β_*) is from 3.15 ± 0.26 M^-1^ to 4.36 ± 0.09 M^-1^ while the corresponding values for the α-carbamate (*K*_α_* = *k*_1α_*/ *k*_*2α*_) are from 7.60 ± 1.75 M^-1^ to 20.25 ± 0.58 M^-1^, at varying ratios of BMAA:HCO_3_^-^ (Figs 2, 3 and Table 1). The equilibrium constants of the β-carbamate are within the range of previously published values [24, 52] while that of the α-carbamate tend to be higher. However, the earlier studies have only focused on the equilibrium process of single carbamate formation while the current approach involves formation/cleavage of two carbamates simultaneously. In this work, in addition to confirming the formation of the carbamate adducts, we demonstrate that BMAA and the α- and β-carbamate adducts simultaneously coexist in the solution with an equilibrium dynamic that is dependent on the concentration of CO_2_.

Nunn and coworkers have studied the formation of BMAA carbamates at neutral conditions [25, 36, 53, 54]. Some of the chemical features that contributes to the carbamate formation in BMAA include, differential degree of ionization of the protons attached to the nitrogen atoms, low pKa of the amino groups [26], and the overall neutral charge at physiological conditions. These features lead to the reaction between the non-protonated α-amino group of BMAA and the bicarbonate which encourages the formation of the carbamate adducts at physiological conditions [36]. Therefore the formation of both α- (2-amino) and β- (3-methylamino) carbamates is a probable explanation of why the BMAA activity is measured only in the presence of bicarbonate/CO_2_ [25, 26, 36, 53]. At equilibrium the relative population of the carbamate adducts depends on the concentration of the bicarbonate ions with the α-carbamate population higher than the β-carbamate (Fig 3). Our data does not show the presence of doubly carbamylated forms of BMAA, which follows a similar observation by Davis et al. [26]. Simultaneous presence of the three molecules in solution further suggests that if either one or both the carbamates are removed from the reaction equilibrium (due to binding to EAA receptors), the equilibrium process will continue to replenish the carbamate populations from free BMAA.

Physiological concentration of bicarbonate ions in bodily fluids range from 20–25 mM [55]. A BMAA concentration of ~5 mM is required for good signal-to-noise ratio particularly for the 2D EXSY experiments. Thus at a BMAA:HCO_3_^-^ ratio of 1:20, the estimated bicarbonate concentration of 100 mM may be physiologically higher. Biological experiments performed in mixed spinal cord cultures to measure motor neuron (MN) loss suggest the concentration dependence of the bicarbonate ions in the media [12, 56]. Therefore, the ratio of BMAA to HCO_3_^-^ might be more important than the absolute concentrations of BMAA or bicarbonate ions themselves. The concentration ratio presented in this work is similar to the experimental conditions of Richter and Mena [13], where the BMAA to bicarbonate ratio was 1 mM to 20 mM. Recently, to investigate the etiopathogenesis of Parkinsonism-dementia (PD), Arif et al. [57] tested the effect of BMAA on protein phosphatase 2A (PP2A) activity and tau hyperphosphorylation in mouse primary neuronal cultures and metabolically active rat brain slices. These experiments are performed typically with 1 mM BMAA and 10 to 35mM of NaHCO_3_, leading to a BMAA: HCO_3_^-^ ratio of 1:10 to 1:35 (at pH 7.4). The BMAA: HCO_3_^-^ ratio in these experiments by Arif et al. [57] are in the same range employed in the results presented in this work.

It must be noted that the measured equilibrium constant (Table 1) depends on the concentration HCO_3_^-^ and therefore the system is still in a *quasi-equilibrium* state. The *quasi*-*equilibrium* constant of the α-carbamate formation shows greater than the β-carbamate with increasing the concentration HCO_3_^-^. Physiological concentrations of BMAA, as low as 10–30 μM, have shown to cause neuronal injury [12, 56]. Thus with a physiological concentration of bicarbonate in the range of 20–25 mM, the HCO_3_^-^ will be > 1,000 times higher than BMAA (BMAA: HCO_3_^-^ = ~1:1,000–1:2,000). Based on the variation in the relative concentrations of the three species in solution (Fig 3), one may assume the concentration of BMAA and the β-carbamate will be the same (~25% each) while the α-carbamate will be twice that of the BMAA (or β-carbamate) with ~50% of the concentration. These estimates are based on the extrapolation of the experimental data (Fig 3) under *in vitro* conditions.

The hypothesized modes of action for BMAA include GluR activation [19] and binding metalloenzymes causing enzyme malfunction [58]. In addition to the different modes of action discussed, the required concentration for neurotoxicity is also varied among studies, yielding conflicting results. BMAA can also cause neurodegeneration through misincorporation into proteins. BMAA has been shown to replace serine within proteins [59], and when BMAA is incorporated the proteins can no longer perform their function due to misfolding. It is unknown if BMAA or the carbamate adducts are misincorporated into proteins. There is a possibility BMAA and the carbamate adducts have different probabilities of misincorporation, and the carbamate adducts might misincorporate to replace different amino acids other than serine. If the carbamate adducts can be misincorporated into proteins in addition to free BMAA, there could be a higher percentage of misfolded or inactive proteins. These misfolded proteins might induce the formation of plaques causing cell damage.

BMAA and HCO_3_^-^ interaction produces two carbamate adducts along with free BMAA in equilibrium at physiological conditions. The concentration of the carbamate adducts are determined with varying BMAA:HCO_3_^-^ ratios, and it was determined that the equilibrium of the three molecules depend on the concentration of CO_2_. With an increased amount of HCO_3_^-^ more of the carbamate adducts are formed. The equilibrium constants governing the formation of the α-carbamate (*K*_α_*) and the β-carbamate (*K*_β_*) and the respective reaction Gibbs free energies (Δ*G*_α_* and Δ*G*_β_*) are determined under equilibrium conditions via 2D proton exchange NMR spectroscopy (EXSY). The α-carbamate tends to be a more preferred adduct of BMAA because the primary amine is more likely to be deprotonated over the secondary amine. Equilibrium carbamate formation is important when determining the neurotoxic concentration of BMAA (directly depends on the physiological conditions of [CO_2_] and pH). This implies a sample with BMAA/HCO_3_^-^ can form a neurotoxic molecule and the neurotoxic concentration is not the initial concentration of BMAA, but a fraction of the initial concentration. The dynamic equilibrium process of BMAA with its carbamate adducts could play a key role in explaining why BMAA can only activate the GluR when in the presence of HCO_3_^-^ and provide insight on BMAA’s mode of action for neurotoxicity.

## Supporting Information

S1 Fig TOCSY spectrum. 400 MHz two-dimensional total correlation (TOCSY) spectra shows the co-existence of BMAA with the primary and secondary carbamate adducts. The reaction mixture consists of 10 mM BMAA and 200 mM bicarbonate in D_2_O. TOCSY experiments were performed at 30°C with a mixing time of 80 ms. The total spectrum is shown in (A), while the sub-spectra in (B), (C) and (D) shows the J-coupling connectivity related to primary carbamate adduct, BMAA (free) and secondary carbamate adduct, respectively.
(PDF)

S2 Fig EXSY spectrum. 400 MHz two-dimensional exchange spectroscopy (EXSY) spectra shows the chemical exchange between the BMAA with the primary and secondary carbamate adducts. The full spectrum is shown in (a) while the expanded region of the αprotons in (B) and the methyl protons in (C). The sample contains 10 mM BMAA and 200 mM bicarbonate in D_2_O. EXSY spectrum is recorded at 30°C and with a mixing time of 400 ms. The BMAA, primary carbamate adduct (α) and the secondary carbamate adduct (β) are marked panels (B) and (C).
(PDF)

S1 Table Example calculations. Spreadsheet used to determine to equilibrium constants from the EXSY spectrum and along with a code (written in R) used eigenvalue calculation (matrix diagonalization).
(PDF)
