# Supplementary figures and images for: Equilibrium Dynamics of β-N-Methylamino-L-Alanine (BMAA) and Its Carbamate Adducts at Physiological Conditions
Source: PLoS One. 2016 Aug 11;11(8):e0160491. doi: 10.1371/journal.pone.0160491 (PMC4981398; doi:10.1371/journal.pone.0160491)

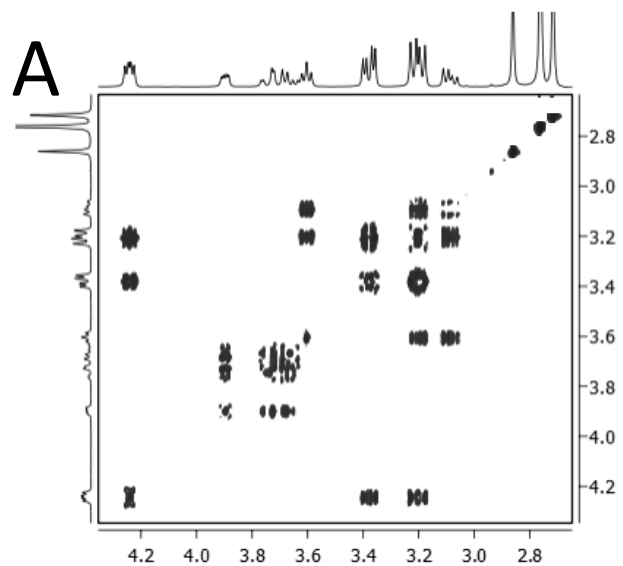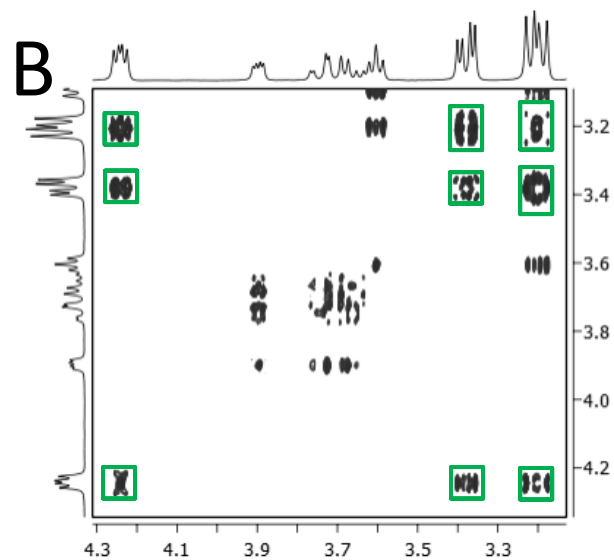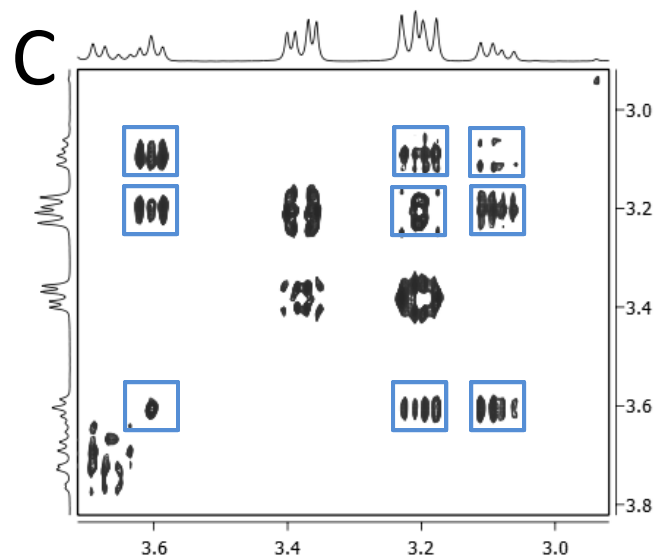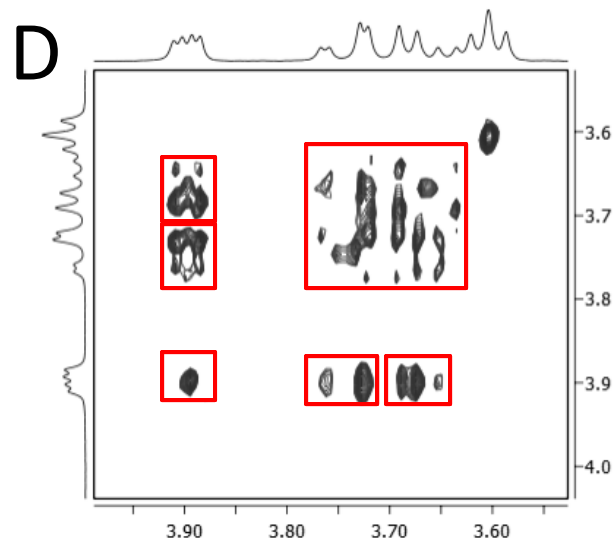

S1 Fig

Supplement: S1 Fig — 400 MHz two-dimensional total correlation (TOCSY) spectra shows the co-existence of BMAA with the primary and secondary carbamate adducts. The reaction mixture consists of 10 mM BMAA and 200 mM bicarbonate in D2O. TOCSY experiments were performed at 30°C with a mixing time of 80 ms. The total spectrum is shown in (A), while the sub-spectra in (B), (C) and (D) shows the J-coupling connectivity related to primary carbamate adduct, BMAA (free) and secondary carbamate adduct, respectively. (PDF) [file pone.0160491.s001.pdf]

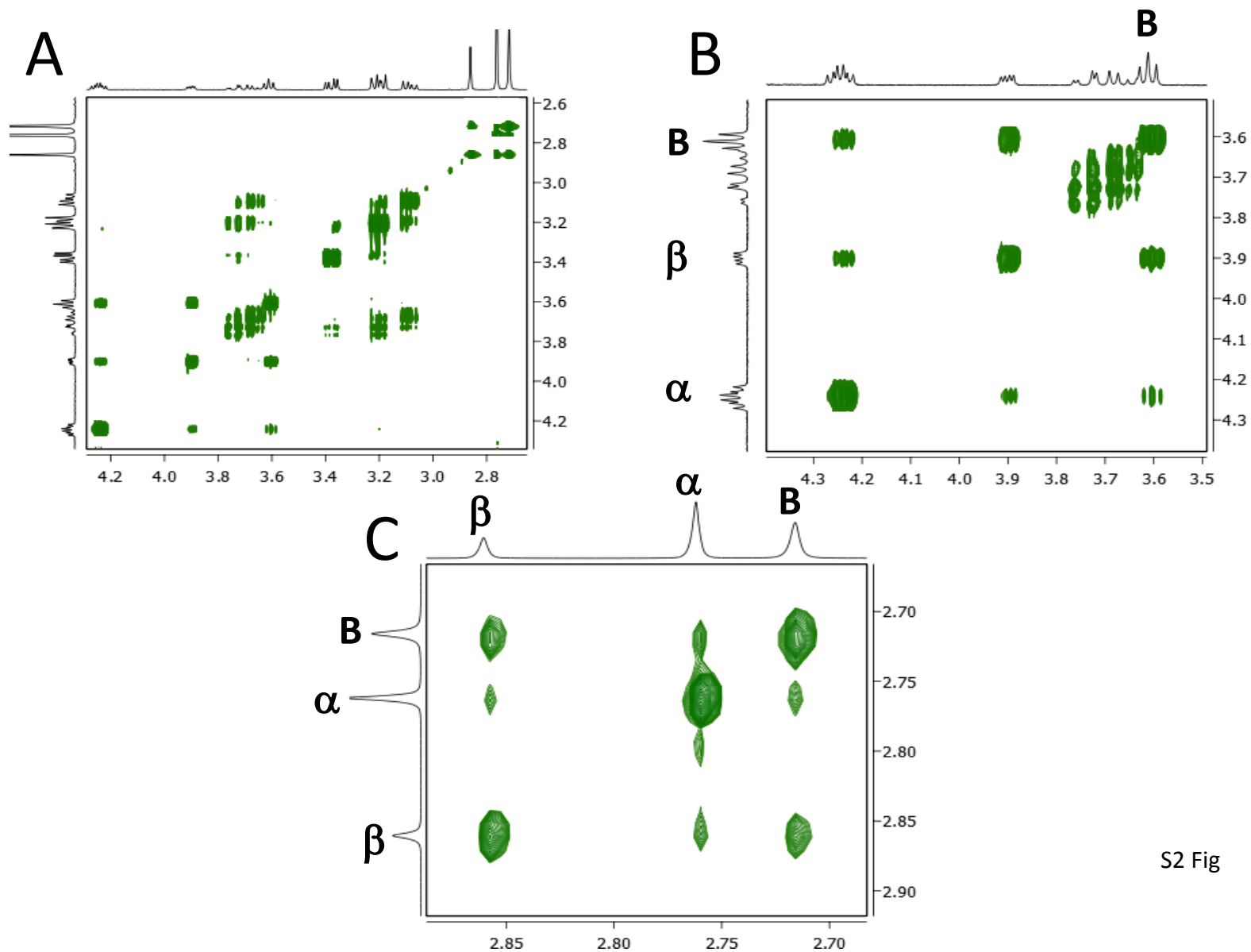

Supplement: S2 Fig — 400 MHz two-dimensional exchange spectroscopy (EXSY) spectra shows the chemical exchange between the BMAA with the primary and secondary carbamate adducts. The full spectrum is shown in (a) while the expanded region of the αprotons in (B) and the methyl protons in (C). The sample contains 10 mM BMAA and 200 mM bicarbonate in D2O. EXSY spectrum is recorded at 30°C and with a mixing time of 400 ms. The BMAA, primary carbamate adduct (α) and the secondary carbamate adduct (β) are marked panels (B) and (C). (PDF) [file pone.0160491.s002.pdf]
