# Supplementary material for: Equilibrium Dynamics of β-N-Methylamino-L-Alanine (BMAA) and Its Carbamate Adducts at Physiological Conditions
Source: PLoS One. 2016 Aug 11;11(8):e0160491. doi: 10.1371/journal.pone.0160491 (PMC4981398; doi:10.1371/journal.pone.0160491)
Supplement: S1 Table — Spreadsheet used to determine to equilibrium constants from the EXSY spectrum and along with a code (written in R) used eigenvalue calculation (matrix diagonalization). (PDF) [file pone.0160491.s003.pdf]

S1 Table

pH 7.6

Peak Intensities from 1D NMR spectrum (AU)

**Sample Conditions**			**Free-BMAA (B)**			**Primary - Carbamate (P)**			**Secondary - Carbamate (S)**		
BMAA	$NaHCO_3$	Temp	Me	α	β	Me	α	β	Me	α	β
5mM	100mM	25C	5.62	2.13		2.38	1	1.04	1.18	0.54	

Peak Volumes from EXSY Experiment

Peaks

	**B**	**P**	**S**
**B**	39.5	2.22	6.57
**P**	2.17	55.73	0.99
**S**	6.93	0.98	16.86

Populations

	**B**	**P**	**S**
**B**	5.62	2.38	1.18
**P**	5.62	2.38	1.18
**S**	5.62	2.38	1.18

A-matrix

	**B**	**P**	**S**
**B**	7.03	0.93	5.57
**P**	0.39	23.42	0.84
**S**	1.23	0.41	14.29

R-marix

	**B**	**P**	**S**
**B**	-3.81	-0.13	-1.11
**P**	-0.05	-6.30	-0.08
**S**	-0.25	-0.04	-5.26

Eqs. 9, 10 & 11

Eq. 12

$$\tilde{A} = \begin{pmatrix} \frac{V_{[BMAA]_T,[BMAA]_T}}{p_{[BMAA]_T}} & \frac{V_{[BMAA]_T,[\alpha-Carbamate]}}{p_{[\alpha-Carbamate]}} & \frac{V_{[BMAA]_T,[\beta-Carbamate]}}{p_{[\beta-Carbamate]}} \\ \frac{V_{[\alpha-Carbamate],[BMAA]_T}}{p_{[BMAA]_T}} & \frac{V_{[\alpha-Carbamate],[\alpha-Carbamate]}}{p_{[\alpha-Carbamate]}} & \frac{V_{[\alpha-Carbamate],[\beta-Carbamate]}}{p_{[\beta-Carbamate]}} \\ \frac{V_{[\beta-Carbamate],[BMAA]_T}}{p_{[BMAA]_T}} & \frac{V_{[\beta-Carbamate],[\alpha-Carbamate]}}{p_{[\alpha-Carbamate]}} & \frac{V_{[\beta-Carbamate],[\beta-Carbamate]}}{p_{[\beta-Carbamate]}} \end{pmatrix}$$

$$\tilde{R} = \begin{pmatrix} R_{BMAA} + k_{1\alpha}^{**} + k_{1\beta}^{**} & -k_{2\alpha}^{*} & -k_{2\beta}^{*} \\ -k_{1\alpha}^{**} & R_{\alpha} + k_{2\alpha}^{*} & -k_{2\alpha\beta}^{*} \\ -k_{1\beta}^{**} & -k_{1\beta\alpha}^{*} & R_{\beta} + k_{2\beta}^{*} \end{pmatrix}$$

	s^{-1}
$R + k_{1P}^{**} + k_{1S}^{**}$	-3.81
k_{2P}^{*}	-0.13
k_{2S}^{*}	-1.11
k_{1P}^{**}	-0.05
$R + k_{2P}^{*}$	-6.30
k_{2PS}^{**}	-0.08
k_{1S}^{**}	-0.25
k_{1SP}^{**}	-0.04
$R + k_{2S}^{*}$	-5.26

Expt. Parameters			units
BMAA	5mM	0.005	M
Na-BC	100mM	0.1	M
pH		7.6	
Temp		298	K
R		8.314462	J/mol.K
pKa (amine)		8.4	
pKa (cabonic)		4.8	

$$\tilde{R} = -\frac{\ln \tilde{A}}{\tau_m} = X(\ln \Lambda) X^{-1} = \begin{pmatrix} R_B + k_{1P}^{**} + k_{1S}^{**} & -k_{2P}^{*} & -k_{2S}^{*} \\ -k_{1P}^{**} & R_P + k_{2P}^{*} & -k_{2PS}^{*} \\ -k_{1S}^{**} & -k_{1SP}^{*} & R_S + k_{2S}^{*} \end{pmatrix}$$

$$k_{1P}^{*} = \frac{k_{1P}^{**}}{['CO_2']}$$

$$k_{1P} = k_{1P}^{*} \frac{['BMAA']['CO_2']}{[BMAA][CO_2]} = k_{1P}^{*} \frac{1}{8.4} \frac{1}{4.8}$$

$$k_{2P} = \frac{k_{2P}^{*}}{[H^{+}]}$$

$$K_P^{*} \qquad K_P^{*} = \frac{k_{1P}^{*}}{k_{2P}^{*}} = \frac{[carbamate]}{['BMAA']['CO_2']}$$

$$\Delta G^{0*} = -RT \ln(K^{*})$$

**Primary**		**Secondary**	
k1**	0.05	0.25	s^{-1}
k1*	0.52	2.46	$M^{-1}s^{-1}$
k1	1.30E-02	6.10E-02	$M^{-1}s^{-1}$
k2*	0.13	1.11	s^{-1}
k2	3.20E-09	2.79E-08	$M^{-1}s^{-1}$
**K***	**4.10**	**2.22**	**M^{-1}**
**ΔG**	**-3.50**	**-1.97**	**$kJ\ mol^{-1}\ K^{-1}$**

```
#
# Matrix routines for eigenvalue calculations
#library(Matrix);

# See the excel sheet for details
#
#
# Using data for BMAA, 1:10 with BMAA 5mM Me region, 30C, pD 7.6
#
# data_1to10 <- matrix(c(23.07020834,  2.795496968,  14.35471317,
#                         2.077091231,   35.94973474,    1.378394458,
#                         4.599084219,   0.8041536,      17.62579491),
#                       nrow = 3, ncol = 3, byrow = TRUE);

data_1to10 <- matrix(c(22.55910717,  2.875305631,       14.32710313,
                       2.031075012,     36.97606397,    1.375743237,
                       4.497195353,     0.827111387,    17.59189324),
                     nrow = 3, ncol = 3, byrow = TRUE);



amat <- data_1to10;
#
mix = 400e-3;
A <- amat; A
X    <- eigen(A)$vectors; X # eigen matrix
Xinv <- solve(X);   Xinv       # inverse eigen matrix
D    <- Xinv %*% A %*% X;  D # Diagolized
#D    <- diag(eigen(A)$values);
logD <- diag(log(eigen(A)$values)); logD
R    <- X %*% logD %*% Xinv; R # Relax matrix
R    <- -1*R/mix;           R;
write.csv(R, file = "data1to10.csv");

mdat <- D;
Remat <- eigen(mdat, symmetric = FALSE);

# Check if it is diagonalized
m <- mdat;
p <- eigen(m)$vectors
d <- diag(eigen(m)$values)
p %*% d %*% solve(p);

amat <- data_1to20;
#
mix = 400e-3;
A <- amat; A
X    <- eigen(A)$vectors; X # eigen matrix
Xinv <- solve(X);   Xinv       # inverse eigen matrix
D    <- Xinv %*% A %*% X;  D # Diagolized
#D    <- diag(eigen(A)$values);
logD <- diag(log(eigen(A)$values)); logD
R    <- X %*% logD %*% Xinv; R # Relax matrix
R    <- -1*R/mix;           R;
write.csv(R, file = "data1to20.csv");
```
